# Different analysis methods of Scottish and English child physical activity data explain the majority of the difference between the national prevalence estimates

**DOI:** 10.1186/s12889-019-6517-7

**Published:** 2019-02-11

**Authors:** Chloë Williamson, Paul Kelly, Tessa Strain

**Affiliations:** 1 Physical Activity for Health Research Centre, Institute of Sport, Physical Education, and Health Sciences, Moray House School of Education and Sport, University of Edinburgh, Edinburgh, UK; 2 MRC Epidemiology Unit, University of Cambridge, Cambridge, CB2 0QQ UK

**Keywords:** Children, Physical activity, Surveillance

## Abstract

**Background:**

The percentages of children in Scotland and England meeting the aerobic physical activity (PA) recommendation differ greatly according to estimates derived from the respective national health surveys. The Scottish Health Survey (SHeS) usually estimates over 70% meeting the recommendation; Health Survey for England (HSE) estimates are usually below 25%. It is plausible that these differences originate from different analysis methods. The HSE monitors the percentage of children in England that undertake 60 min of moderate-to-vigorous PA on each day of the week (‘Daily Minimum Method’ (DMM)). The SHeS monitors the proportion that undertakes at least seven sessions of moderate-to-vigorous PA, with an average daily duration ≥60 min in Scotland (‘Weekly Average Method’ (WAM)). We aimed to establish how much this difference in analysis methods influences prevalence estimates.

**Methods:**

PA data from 5 to 15 year olds in the 2015 HSE and SHeS were reanalysed (weighted *n* = 3840 and 965, respectively). Two comparable pairs of estimates were derived: a DMM and WAM estimate from the HSE not including travel to/from school, and WAM estimates from the HSE and the SHeS including travel to/from school. It is not possible to calculate a DMM estimate from the SHeS due to questionnaire design. Results were presented for the total samples, and by sex and age sub-groups.

**Results:**

The HSE WAM estimate was 31.7 (95% CI: 30.2–33.3) percentage points higher than the DMM estimate (54.3% (95% CI: 52.6–56.0) and 22.6% (95% CI: 21.2–24.1) respectively). The magnitude of this difference differed by age group but not sex. When comparable WAM estimates were derived from the SHeS and the HSE, the SHeS was 11.8 percentage points higher (73.6% (95% CI: 69.8–77.1) and 61.8% (95% CI: 60.2–63.5) respectively). The magnitude of this difference differed by age group and sex.

**Conclusions:**

The results indicate that the difference in the analysis method explains the majority (approximately 30 percentage points) of the difference in the child PA prevalence estimates between Scotland and England (leaving approximately 12 percentage points representing true differences or related to questionnaire differences). These results will help national surveillance determine how to increase comparability between the U.K. home nations.

## Background

There are a number of surveys within the UK home nations that estimate the proportion of children meeting the aerobic physical activity (PA) recommendation (≥60 min of moderate-to-vigorous PA (MVPA) every day [1]). For example, the national health surveys and the Health Behaviour in School-Aged Children survey (HBSC) [2–4]. Cross-country comparisons are inevitable, indeed often encouraged [5]. The study designs, measurement instruments, methods of administration, and age ranges included vary between surveys, but most estimate between 10 and 30% of children in their target population meet the 60 min threshold [2, 4, 6, 7]. The Scottish Health Survey (SHeS) is an exception to this, having reported estimates of over 70% annually since 2008 [8]. The conclusion that Scottish children are more active than those in other home nations is not supported when the Scottish, English and Welsh HBSC survey results are compared [4, 6, 9]. The comparable age and sex sub-group prevalence estimates from the different nations’ HBSC surveys are all within six percentage points of each other, ranging between 11 and 30% [4, 6, 9].

A plausible contributing factor is that the SHeS estimates are for 2–15 year olds, while most others are for older age groups (e.g. 5–15 year olds in Health Survey for England (HSE), or 11–16 year olds in the HBSC surveys). This may inflate the SHeS estimates because the youngest are the most active age group, although the evidence suggests it would account for a few percentage points at most [8]. There is a separate issue around whether 2–4 year olds should be included in the prevalence estimate given that the recommendation in question applies to 5–18 years olds [1]. Those under 5 years that can walk unaided are recommended to be physically active for at least 3 h per day [1]. The reason for their inclusion in the SHeS estimates is to maintain the trend data that began before a distinction was made between the age groups.

Some have attributed the high prevalence figures to the SHeS questionnaire over-estimating MVPA time [10]. This line of thought is based on a convergent validity study comparing the SHeS questionnaire^1^ to uniaxial waist-worn accelerometers in 130 6–7 year olds [11]. The estimates for total daily MVPA derived from the questionnaire were approximately two hours higher than those from accelerometry. As the SHeS does not specifically explain to respondents that they should only report activity of at least moderate intensity [12], it is easy to see how these conclusions are drawn.

However, the overwhelming majority of activities that are prompted are considered by the Youth Compendium of Physical Activities to be of at least moderate intensity (e.g. sweeping leaves, running about, football; [13]). Also, even if there were some light intensity activities reported, one would expect there to be a comparable degree of reporting in the HSE where the same types of activities are prompted, just under different categories and in a slightly different order (see Table 1) [14]. This does not appear to be the case as there was still a difference of over 50 percentage points between the 2015 estimates (73% in SHeS and 22% in HSE) [2, 3].

**Table 1:** The measurement of child physical activities in the 2015 SHeS and 2015 HSE

Specific physical activity	SHeS 2015 category of activity	HSE 2015 category of activity	Included in estimates presented in this study			
			(1) HSE DMM no school travel estimate^a^	(2) HSE WAM no school travel estimate	(3) HSE WAM all activity estimate	(4) SHeS WAM estimate
Activity as part of school lessons	School based activity	School lessons	✓	✓	✓	✓
Walking to school	Walking	Travel to school^b^	X^b^	X	✓	✓
Cycling to school	Sports and exercise activities		X^b^	X	✓	✓
Walking (not to school)	Walking	Informal activities	✓	✓	✓	✓
Active play activities	Active play		✓	✓	✓	✓
Heavy housework/gardening	Housework^c^		✓	✓	✓	✓^c^
Sport and exercise activities	Sport and exercise activities	Formal activities	✓	✓	✓	✓
Any other activities similar to those prompted under active play and/or sport and exercise activities	Active play	Other informal/formal activities	✓	✓	✓	✓

*SHeS* Scottish Health Survey, *HSE* Health Survey for England, *WAM* Weekly Average Method, *DMM* Daily Minimum Method (see article text for descriptions). a: any minor differences between this estimate and the published headline HSE figures are because those not at school are allocated 0 for school lesson activity rather than being excluded; b: the HSE does not ask the specific days (only number of days) on which the child either walked or cycled to, or part of the way to, school/nursery/playgroup. This means it cannot be included in any prevalence estimate using the DMM; c: only asked of those aged 8–15 years

**Fig. 1:**
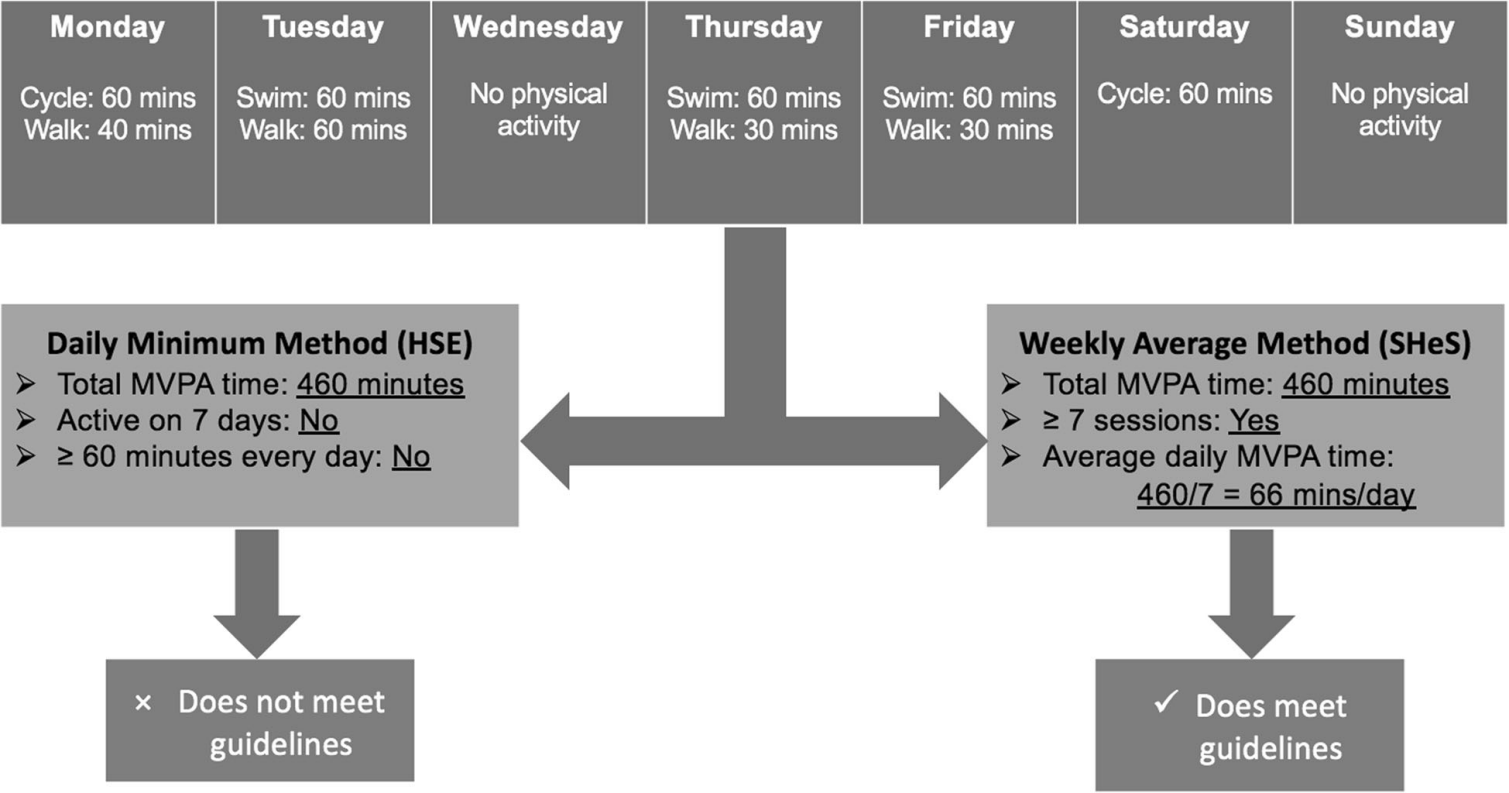
Demonstration of how the use of ‘Daily Minimum Method’ or the ‘Weekly Average Method’ may lead to different results from the same data

Given that the SHeS and HSE questionnaires are so similar, yet produce such different prevalence estimates, we investigated how the data were processed. A subtle but potentially important difference between the SHeS and HSE surveys is how the frequency of activities are reported, which has implications for how the data are processed. In the SHeS, children or their parents are asked to report how many *sessions* of each activity they have done in the previous seven days [12], making it impossible to ascertain whether child respondents to the SHeS achieve ≥60 min of MVPA on each specific day. In order to estimate the frequency element of the recommendation, the SHeS makes the assumption that the first 7 sessions are carried out on different days. Recommendation compliance is then estimated by the proportion of children that report ≥7 sessions, and whose total weekly duration is ≥420 min (7*60). For the rest of this paper, we use the term ‘Weekly Average Method’ (WAM) to describe this approach. In the HSE, activities are reported for specific days in the previous seven [14], and a child is judged to meet the recommendation if they report ≥60 min per day on each specific day of the preceding week (the ‘Daily Minimum Method’ (DMM); see Fig. 1). The DMM is therefore a mathematically stricter version of the WAM, additionally requiring the child to have been active on every single day and to have undertaken ≥60 min on each day. This stricter threshold will always lead to lower prevalence estimates.

**Fig. 2:**
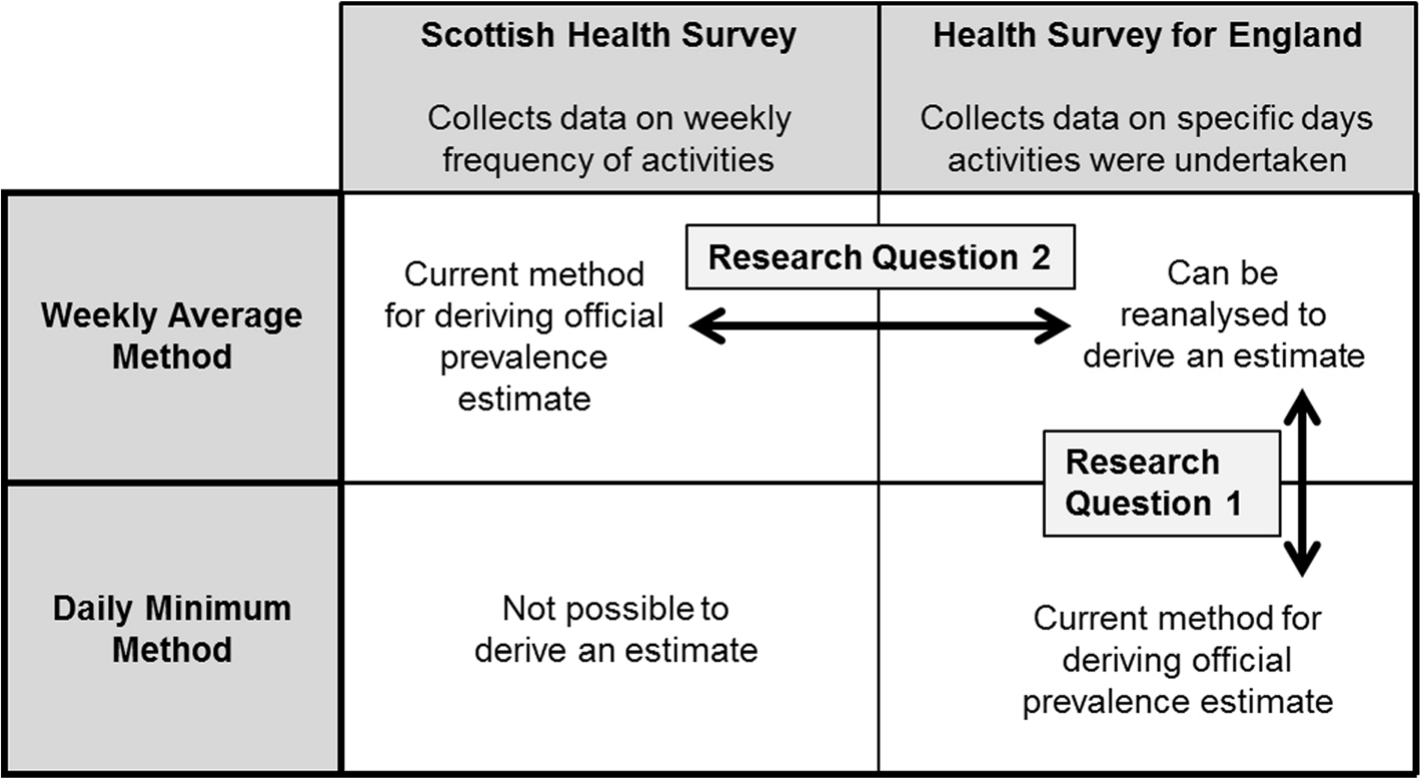
Schematic overview of what analysis methods are possible in the Scottish Health Survey and Health Survey for England, and the research questions of the study

We hypothesise that it is this difference in analysis method that is the key driver behind the differences in the SHeS and HSE prevalence estimates. We feel it is important to quantify the magnitude of this difference because it will offer strategies for appropriate changes to be made to surveillance methods to increase comparability between home nations. It is timely as such changes may be precipitated by the 2018 update of the CMO PA Recommendations [15]. We will test this hypothesis by reanalysing HSE and SHeS data to generate comparable estimates between Scotland and England. Figure 2 provides an overview of what methods (WAM or DMM) are possible in the two surveys, and what comparisons can be drawn.

Our two research questions are: (1) what is the percentage point difference between comparable WAM and DMM estimates for England? (2) What is the percentage point difference between comparable WAM estimates for Scotland and England? These differences will be assessed for all children, and by age and sex sub-groups.

## Methods

### Data sources

The study was approved by the Moray House School of Education ethics committee and all authors with access to the data agreed to the UK Data Archive End User Licence. The 2015 HSE and SHeS datasets were downloaded from the UK Data Archive on 6th April 2018 [16, 17], previous preliminary work was undertaken on the respective 2012 datasets. Both surveys are sampled so that they are nationally representative of their respective national populations that live in private households after weighting on key demographic characteristics; further details are in the surveys’ technical reports [12, 14].

### Sample

The sample age range was restricted to 5–15 year olds. Those younger have different PA guidelines [1]. Those older for whom the recommendation does apply (16–18 year olds) are treated as adults by both surveys and so responded to a different questionnaire. Those responding ‘don’t know’ or who refused to answer a question were excluded (< 2%). Those not at school were included in all analyses but were allocated zero time/frequency for any activity at or involved in the travel to/from school. This left 4096 respondents to the 2015 HSE and 946 respondents to the 2015 SHeS with complete PA data.

### Physical activity measurement

Table 1 shows that the HSE and SHeS ask about the same activities but that they are split under different categories. This means the ordering is slightly different. Both surveys have a recall period of the seven days prior to interview. The full questionnaire transcripts are published in the technical reports [12, 14]. Parents answered on behalf of children aged 5–12, and children aged 13–15 answered themselves.

Four summary measures of compliance to the PA recommendation were derived (see Table 1):

- (1) from the 2015 HSE using the DMM. This did not include travel to/from school because these data were reported as a weekly frequency rather than on specific days.
- (2) From the 2015 HSE using the WAM, also excluding travel to/from school such that it was comparable with (1).
- (3) From the 2015 HSE using the WAM, including travel to/from school as a weekly frequency was not a limitation for this method.
- (4) From the 2015 SHeS using the WAM, including all activity so it was comparable with (3).

**Fig. 3:**
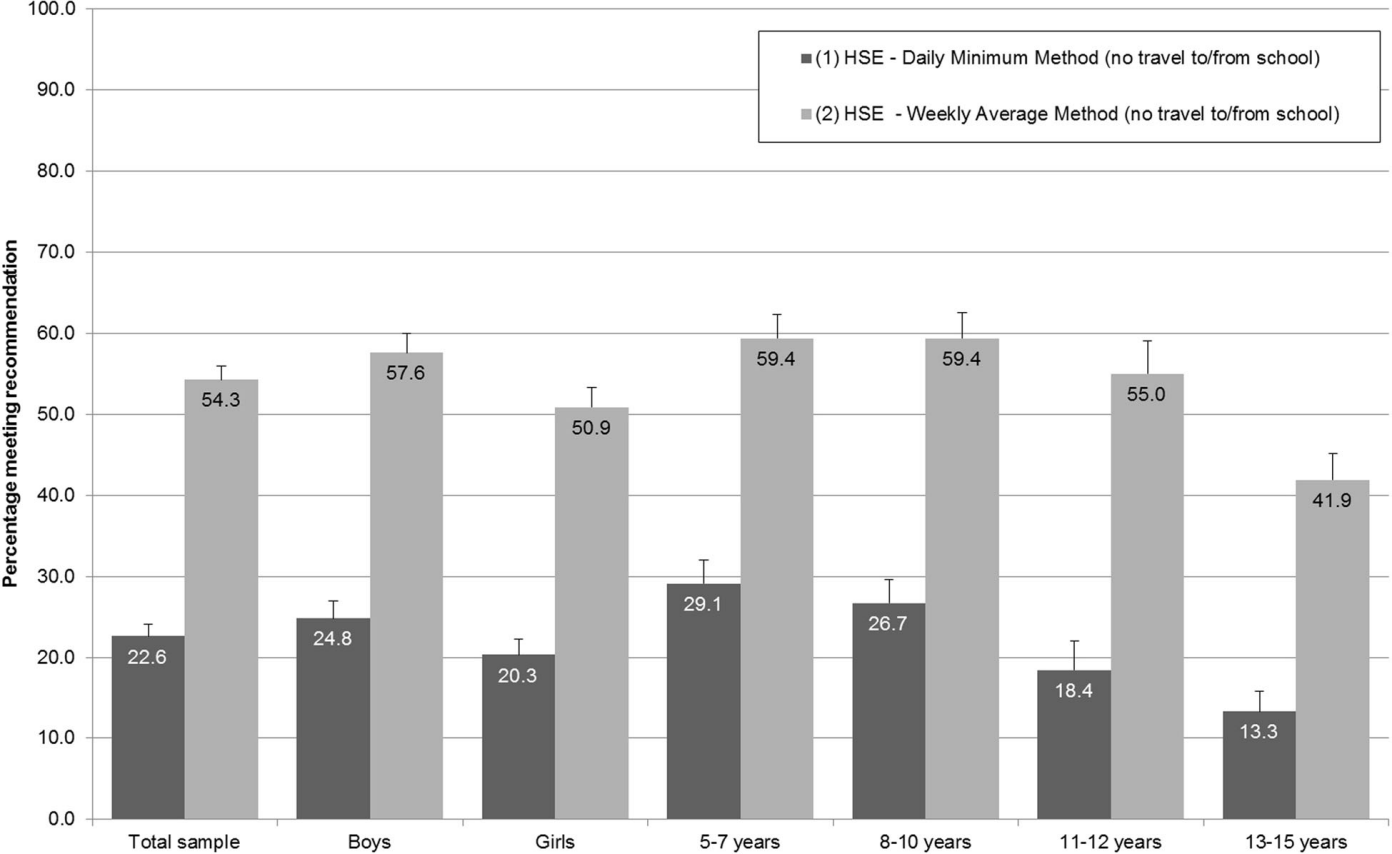
The percentages of children in the 2015 Health Survey for England meeting the aerobic physical activity recommendation according to the analysis methods

**Fig. 4:**
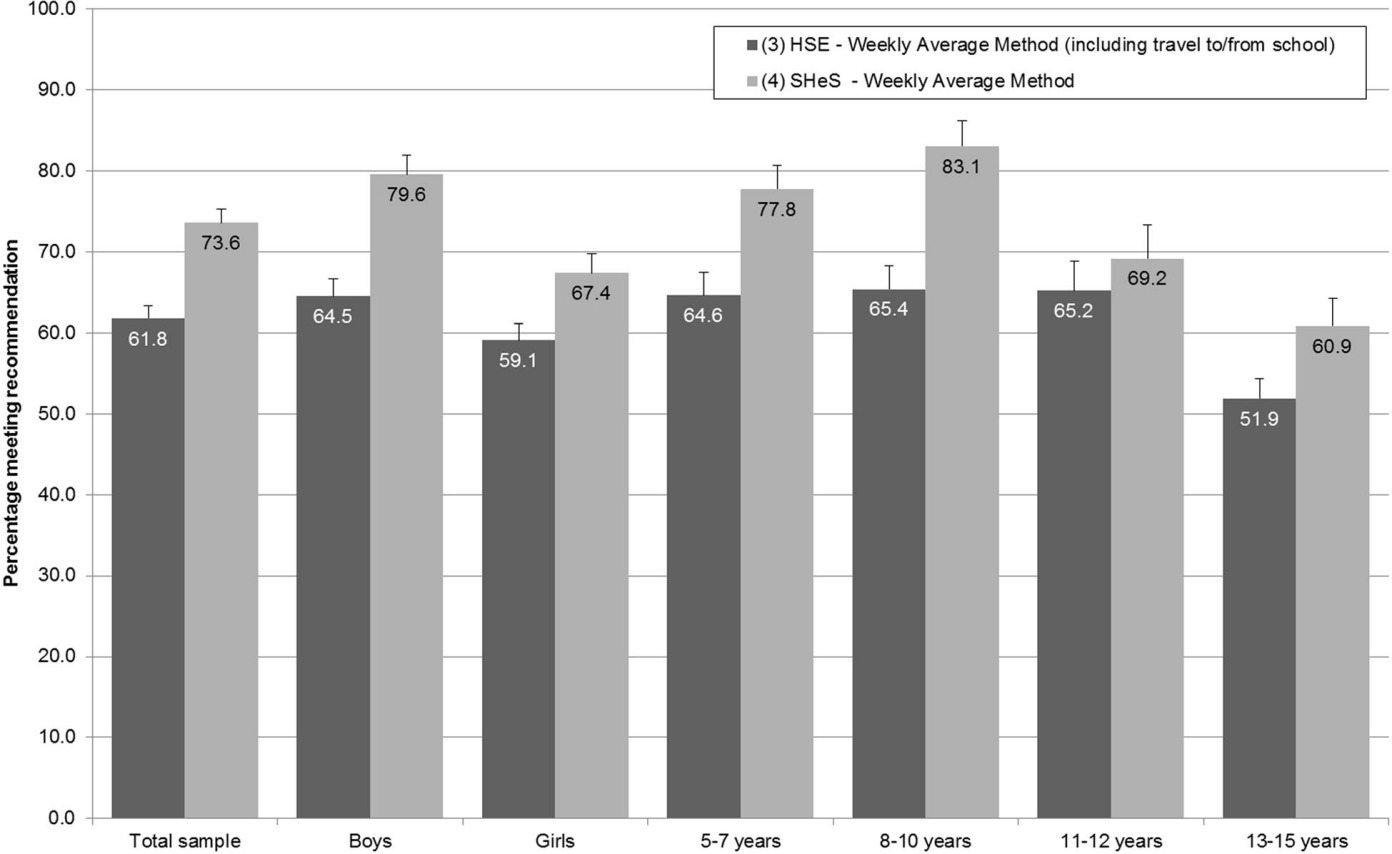
The percentages of children in the 2015 Health Survey for England and the 2015 Scottish Health Survey meeting the aerobic physical activity recommendation according to the different analysis methods

The mean differences between the pairs of estimates (2)–(1) (research question 1 on Fig. 2) and (4)–(3) (research question 2 on Fig. 2) were calculated. The first comparison indicated the magnitude of the difference that the analysis method makes, because it uses the two methods in the same sample. The second comparison was between the WAM estimates from the two different surveys. This was an indication of how much of the difference was not explained by the analysis method (possibly true difference, or other methodological differences that could not be standardised).

Statistical differences by sex and age group (5–7, 8–10, 11–12, 13–15 years) were assessed using adjusted Wald F-tests. All estimates were weighted to account for non-response and selection bias. Analyses were carried out using Stata/SE v14.2, Texas, USA.

## Results

The weighted sample sizes were *n* = 3840 and *n* = 965, for the 2015 HSE and SHeS respectively (see Table 2). The breakdown of the samples by sex and age group were near identical (< 2 percentage point differences).

**Table 2:** Descriptive characteristics of the analysis samples from each survey

	HSE 2015 sample		SHeS 2015 sample	
	weighted n	%	weighted n	%
Total sample	3840	100	965	100
Boys	1958	51.0	493	51.1
Girls	1881	49.0	472	48.9
5–7 years	1133	29.5	281	29.1
8–10 years	1058	27.5	272	28.2
11–12 years	723	18.8	167	17.4
13–15 years	927	24.1	244	25.3

When the 2015 HSE sample was analysed according to the DMM and the WAM (both not including travel to/from school), the WAM estimate was 31.7 percentage points higher than the DMM estimate (22.6% (95% CI: 21.2–24.1) and 54.3% (95% CI: 52.6–56.0); see Fig. 3, Table 3). An increase of comparable magnitude was evident in both boys and girls (*p* > 0.05 for difference between sexes) but differed by age group (*p* < 0.05). The differences between the estimates for the youngest two age groups (5–7 year olds and 8–10 year olds) were within two percentage points of the sample mean. The difference was highest for 11–12 year olds (36.6 percentage points (95% CI: 32.7–40.6)) and lowest for 13–15 year olds (28.5 (95% CI: 25.5–31.6)).

**Table 3:** The mean percentage point differences and 95% confidence intervals between comparable estimates

	(1) HSE – DMM no travel to/from school)	(2) HSE - WAM (no travel to/from school)	Mean difference: (2) HSE WAM – (1) HSE DMM	(3) HSE – WAM (including travel to/from school)	(4) SHeS - WAM	Mean difference: (4) SHeS WAM – (3) HSE WAM
	% (95% CI)	% (95% CI)	Percentage point (95% CI)	% (95% CI)	% (95% CI)	Percentage point (95% CI)
Total sample	22.6 (21.2, 24.1)	54.3 (52.6, 56.0)	31.7 (30.2, 33.3)	61.8 (60.2, 63.5)	73.6 (69.8, 77.1)	11.8 (8.3, 15.3)
Boys	24.8 (22.8, 27.0)	57.6 (55.2, 60.0)	32.8 (30.6, 35.0)	64.5 (62.2, 66.8)	79.6 (75.0, 83.5)	15.0 (10.6, 19.4)
Girls	20.3 (18.4, 22.3)	50.9 (48.5, 53.3)	30.6 (28.4, 32.8)	59.1 (56.7, 61.4)	67.4 (61.3, 73.0)	8.3 (3.0, 13.7)
			*p > 0.05*			***p < 0.05***
5–7 years	29.1 (26.4, 32.0)	59.4 (56.3, 62.3)	30.3 (27.5, 33.0)	64.6 (61.6, 67.4)	77.8 (71.8, 82.9)	13.2 (7.4, 19.1)
8–10 years	26.7 (23.9, 29.6)	59.4 (56.3, 62.5)	32.8 (29.7, 35.8)	65.4 (62.3, 68.4)	83.1 (77.7, 87.5)	17.7 (12.1, 23.3)
11–12 years	18.4 (15.3, 22.0)	55.0 (50.8, 59.1)	36.6 (32.7, 40.6)	65.2 (61.1, 69.1)	69.2 (59.6, 77.3)	4.0 (−5.5, 13.4)
13–15 years	13.3 (11.2, 15.8)	41.9 (38.6, 45.2)	28.5 (25.5, 31.6)	51.9 (48.5, 55.3)	60.9 (53.0, 68.2)	9.0 (1.3, 16.6)
			***p < 0.05***			***p < 0.05***

*HSE* Health Survey for England, *SHeS* Scottish Health Survey, *WAM* Weekly Average Method, *DMM* Daily Minimum Method (see article text for descriptions). *P*-values refer to the testing of the null hypotheses that the proportion is equal across sex and age sub-groups

When the comparable estimates (i.e. including all reported activities) were derived from the 2015 HSE and the 2015 SHeS using the WAM, there was an 11.8 percentage point (95% CI: 8.3–15.3) difference (61.8% (95% CI: 60.2–63.5) for the HSE and 73.6% (95% CI: 69.8–77.1) for the SHeS; see Fig. 4, Table 3). This difference was greater amongst boys than girls (15.0 percentage points (95% CI: 10.6–19.4) and 8.3 (95% CI: 3.0–13.7) respectively; *p* < 0.05). The difference was highest amongst 8–10 year olds (17.7 percentage points (95% CI: 12.1–23.3)) and lowest amongst 11–12 year olds (4.0 (95% CI: -5.5-13.4); *p* < 0.05 for difference between age groups).

## Discussion

These findings show that small differences in the analysis method can lead to a critical difference in prevalence. Our results indicate the majority of the difference between the SHeS prevalence estimate and those from other UK national surveys is due to a different interpretation of the ‘60 minutes per day’ recommendation. This is shown by the 31.7 percentage point increase (95% CI: 30.2–33.3) in prevalence when the HSE sample was reanalysed according to the WAM rather than the DMM. There remains, however, an 11.8 percentage point (95% CI: 8.3–15.3) difference between the comparable WAM estimates from the SHeS and the HSE. This may in part be due to a true difference between the PA levels of children in Scotland and England, but may also be due the different categorisation of activities and ordering of questions in the SHeS and the HSE.

The largest difference between the DMM and WAM estimates was in 11–12 year olds and the lowest in 13–15 year olds. By definition, the differences occur when children undertake a total ≥ 420 min/week but not divided evenly throughout the week. Therefore, a potential reason for the high difference amongst 11–12 year olds is that this is a transitional age where children move from primary to secondary school. This might mean a shift from daily informal PA, such as active play at lunchtime, to longer but less frequent bouts of PA such as gym sessions or after-school sports. The smallest difference amongst 13–15 year olds may be explained by low total weekly durations, meaning neither the DMM nor WAM definition of “active” was met. Future research could investigate patterns through which PA is accumulated in different age groups to establish why some are more affected by a difference in analysis methods than others.

Our results lead to an important conclusion; small differences in analysis methods could invalidate cross-country comparisons and at worst will lead to misguided policy and practice. This highlights the need for caution when interpreting the results of cross-country comparison projects such as the Active Healthy Kids Report Cards and the GoPA! country cards [5, 18]. While they may serve a political or advocacy purpose, any interpretation that country Y has a higher/lower PA prevalence than country Z should be approached with caution. We should question: how were data collected and analysed, before we ask: are prevalence estimates real and why do they exist?

This naturally leads to the question: what is the most appropriate interpretation of the guideline? The Technical Report accompanying the 2011 UK PA guidelines is clear that they intended the DMM interpretation [19]. The recommendation for daily physical activity was included to ‘promote a pattern of regular PA’ and to assist communication of a simple message. However, they also state that ‘there is no evidence that missing one day (or two or three days) in a week has any measurable negative effect on health’ [19]. However, there is room for discussion over the evidence used to support this decision. It may be that a “rest day” each week allows for better recovery, rest and physiological adaption if sufficient activity is completed over 7-day period. Importantly it may also be better for affect and enjoyment to take a break; the potential impact on long term habit formation is not clear.

In light of this, we call on developers of PA recommendations to also include guidance on how to collect, process and analyse data. An example of this comes from Canada where specific surveillance guidance was provided to accompany the new 24-h PA guidelines for children and youth [20]. This should be considered a necessary step in the implementation of any recommendations. Our results show that even small room for interpretation or “researcher or analytical degrees of freedom” can lead to huge differences in prevalence estimates. This is likely to be particularly relevant in future should device-based measures be introduced, given the permutations in processing and analysis [21, 22]. The UK PA guidelines are being reviewed this year, and, for the first time, there is a specific sub-group to consider implementation and surveillance issues. This is a useful opportunity to make sure that the surveillance methods are able to implement the guidelines as intended.

These results are comparable to Scottish and Canadian studies that analysed accelerometry data using two analytical approaches: the DMM as defined in this study and a weekly total ≥ 420 min [23, 24]. The DMM produced estimates of 11% in Scotland and 2.9–6.2% in Canada across different survey years. The weekly total approach produced estimates of 68% and 28.8–41.0%, respectively. Taken together, they show that it is important not to conflate the issues of whether device-based surveillance of child PA should be introduced in Scotland with the issues of the high prevalence estimates. Whilst there may be many other relevant considerations for changing national surveillance of child PA to accelerometry, generating comparable prevalence figures to the other national surveys within the UK could be achieved through changes to the questionnaire and/or analysis method.

The strengths of this study are that it is the first to attempt to directly address the issue of why the prevalence figures are so different between Scotland and England. Through the research questions posed and the study design chosen, we were able to isolate the issue of the recommendation interpretation from other persisting concerns around the validity and reliability properties of the measurement instruments and the different age ranges used in the national prevalence estimates. Also, the large effect size increases confidence in the conclusions. Early findings of this work were shared with the Scottish Government and those running the SHeS, and have been part of considerations around changes to the measurement instrument. They will continue to do so as the UK PA guidelines are re-evaluated for publication in 2019. The findings have also relevance for a wider audience: Jurakic and Pedišić (2012) [25] showed that differences in recommendation interpretation occur beyond the U.K.

The main limitation of this study was that we were unable to eliminate all differences between the questionnaires, which may have affected the comparisons between the HSE and SHeS WAM estimates. It is also possible that the requirement of the HSE to report activity on specific days made it somewhat like a diary, leading respondents to answer in a different manner to those in the SHeS sample. It has been argued that diaries improve recall and reduce the potential for social desirability bias compared to general questionnaires, as stronger associations with estimates derived from doubly labelled water have been demonstrated [26]. We also chose to focus entirely on the issue of analytical approach and so have not quantified the effect of other differing factors in the published national prevalence figures such as differing age ranges. We were also not able to account for the stratified and clustered sampling procedures when calculating the 95% CIs because of the small number of primary sampling units in some HSE strata. Therefore, the true variance on the estimates may be fractionally larger than those presented.

## Conclusions

In conclusion, this study has demonstrated that it is the difference in analysis method that explains the majority of the difference in the child PA prevalence estimates between Scotland and England. These results will help those involved in national surveillance determine how to increase comparability between the U.K. home nations.

## Abbreviations

DMM: Daily minimum method
HBSC: Health behaviour in school-aged children
HSE: Health survey for England
MVPA: Moderate-to-vigorous physical activity
PA: Physical activity
SHeS: Scottish health survey
WAM: Weekly average method

## Footnotes

1 At the time, this was also the HSE questionnaire. The HSE questionnaire subsequently changed (in 2008) the way activities were grouped into categories and the ordering of questions. It also changed to ask on which specific day activities were undertaken, rather than a weekly frequency.
